# THE ASSOCIATION OF LIFESTYLE AND SOCIAL ENGAGEMENT WITH DEMENTIA: A NATIONWIDE POPULATION-BASED COHORT STUDY

**DOI:** 10.1093/geroni/igac059.2791

**Published:** 2022-12-20

**Authors:** Jiyoon Jang, Sang Hwa Lee, Eunhee Cho

**Affiliations:** College of Nursing, Yonsei University, Seoul, Seoul-t’ukpyolsi, Republic of Korea; College of Nursing, Yonsei University, Seoul, Seoul-t’ukpyolsi, Republic of Korea; College of Nursing, Yonsei University, Seoul, Seoul-t’ukpyolsi, Republic of Korea

## Abstract

As the aging population increases, the number of people with dementia is expected to rise rapidly, resulting in a growing burden on families and society. Delaying and preventing the clinical onset of dementia are significant public health goals. Unhealthy lifestyles and social disengagement have been identified to be modifiable risk factors for all-cause dementia. However, this has not been fully examined in the Korean population. Therefore, this study aimed to determine the association of lifestyle and social engagement with dementia incidence, based on the Korean Longitudinal Study on Aging (KLoSA) database with recently added dementia-related variables (diagnosis, medication administration, and treatment). The study included 5,071 participants; cases are patients diagnosed with dementia from 2010 to 2020 and controls are older adults without dementia. Logistic regression analysis was used to examine the relationship of dementia with lifestyle factors (alcohol consumption, smoking, exercising) and social engagement. Among the lifestyle factors, the current smoking status was associated with an increased risk of dementia (OR = 2.359, p < .05) after controlling the health-related covariates and general characteristics. Furthermore, increased participation in social activities was significantly related with a decreased risk of dementia (OR = 0.575, p < .01). This study highlighted the importance of non-smoking and social engagement in reducing the risk of dementia in later life. Therefore, implementation of interventions that focus on these leading risk factors can significantly reduce the burden of dementia in Korea.

